# Suppression of diabetic retinopathy with GLUT1 siRNA

**DOI:** 10.1038/s41598-017-07942-x

**Published:** 2017-08-07

**Authors:** Zhi-Peng You, Yu-Lan Zhang, Ke Shi, Lu Shi, Yue-Zhi Zhang, Yue Zhou, Chang-yun Wang

**Affiliations:** Department of Ophthalmology, The Second Affiliated Hospital, Nanchang University, Nanchang, 330006 China

## Abstract

To investigate the effect of glucose transporter-1 (GLUT1) inhibition on diabetic retinopathy, we divided forty-eight mice into scrambled siRNA, diabetic scrambled siRNA, and GLUT1 siRNA (intravitreally injected) groups. Twenty-one weeks after diabetes induction, we calculated retinal glucose concentrations, used electroretinography (ERG) and histochemical methods to assess photoreceptor degeneration, and conducted immunoblotting, leukostasis and vascular leakage assays to estimate microangiopathy. The diabetic scrambled siRNA and GLUT1 siRNA exhibited higher glucose concentrations than scrambled siRNA, but GLUT1 siRNA group concentrations were only 50.05% of diabetic scrambled siRNA due to downregulated GLUT1 expression. The diabetic scrambled siRNA and GLUT1 siRNA had lower ERG amplitudes and ONL thicknesses than scrambled siRNA. However, compared with diabetic scrambled siRNA, GLUT1 siRNA group amplitudes and thicknesses were higher. Diabetic scrambled siRNA cones were more loosely arranged and had shorter outer segments than GLUT1 siRNA cones. ICAM-1 and TNF-α expression levels, adherent leukocyte numbers, fluorescence leakage areas and extravasated Evans blue in diabetic scrambled siRNA were higher than those in scrambled siRNA. However, these parameters in the GLUT1 siRNA were lower than diabetic scrambled siRNA. Together, these results demonstrate that GLUT1 siRNA restricted glucose transport by inhibiting GLUT1 expression, which decreased retinal glucose concentrations and ameliorated diabetic retinopathy.

## Introduction

Diabetic retinopathy (DR) is one of the most common complications of diabetes mellitus (DM). DR often results in decreased vision and even blindness caused by macular edema, retinal detachment, and vitreous hemorrhage. The number of patients with diabetes may grow to 642 million in 2040^1^. DR has been recognized as a microangiopathy, as well as a neurodegenerative disease. Although the detailed mechanism underlying DR is unclear, two major global multicenter studies on diabetes, DCCT^2^ and UKPDS^3^, have revealed that a long-term high blood glucose level is the decisive factor for DR development. Moreover, excessive generation of retinal oxidative stress products^4^, activated protein kinase C^5^, and increased synthesis of glycosylated end products^6^ under the environment of high blood glucose levels initiate the impairment of retinal tissues and cells^4^. Since lesions are induced by high blood glucose levels, we hypothesize that DR progression can be relieved by restricting glucose transfer into the retina, thereby decreasing its local glucose content. Glucose transporter-1 (GLUT1) is the only currently known carrier of glucose through the blood–retinal barrier and is also responsible for the distribution of glucose in ganglion cells, photoreceptor cells, and Müller cells in the retina; GLUT1 is primarily expressed in the vascular endothelial cells of the inner blood–retinal barrier and retinal pigment epithelial cells of the outer blood–retinal barrier^7^. GLUT1 was identified as a promising target for diabetic retinopathy^8^, but current research did not observe particular effect on retinopathy including neuron degeneration and microangiopathy with means of GLUT1 downregulation.

In this study, we intend to assess and compare electroretinography (ERG) amplitudes, outer nuclear layer (ONL) thicknesses, and cone cell densities in diabetic mice. The results will be used to determine pathological changes in photoreceptor cells, measure the expression levels of retinal inflammatory factors, quantify adherent leukocytes in retinal vessels and determine the leakage area of the inner blood–retinal barrier to compare the level of microangiopathy. Our purpose is to investigate the effect of GLUT1 negative regulation on retinopathy via the above parameters to verify whether suppression of GLUT1 would be benefit for DR.

## Results

### Establishment of the diabetic model and measurement of body weight and blood glucose levels in the three groups

At 7 d after intraperitoneal injections with streptozotocin, all blood glucose levels of the 48 males C57BL/6 mice (diabetic scrambled siRNA and GLUT1 siRNA groups) used for the establishment of the diabetic model were greater than 300 mg/dL, and the success rate of modeling was 100%. The body weight and blood glucose levels of the mice were measured again at 20 weeks after the diabetic model was established. No significant differences in the body weights of the mice were found among the three groups when the diabetic model was successfully established. However, the body weight of the scrambled siRNA group was significantly higher than that of the diabetic scrambled siRNA and GLUT1 siRNA groups by 40.44% and 35.59%, respectively, at 20 weeks after the diabetic model was established (P < 0.01). Both groups with diabetes exhibited an emaciated body, whereas their water intake, food intake, and urine volume were higher than those of the scrambled siRNA group. At two time points: 1 d and 20 weeks after the diabetic model establishment the blood glucose levels of the scrambled siRNA group were lower than those of the diabetic scrambled siRNA group by 46.85% and 55.37%, respectively. The blood glucose levels were significantly lower than those of the GLUT1 siRNA group by 47.36% and 54.39% (P < 0.05). However, no significant difference was found in the blood glucose levels between the diabetic scrambled siRNA and GLUT1 siRNA groups at both time points (Table 1).

**Table 1:** Body weight and blood glucose levels of the three groups (*n* = 16, $\overline{x}$ ± S).

group	body weight (g)		blood glucose level (mg/dL)	
	at 1 d after the diabetic model was established	at 20 weeks after the diabetic model was established	at 1 d after the diabetic model was established	at 20 weeks after the diabetic model was established
Scrambled siRNA	24.51 ± 2.13	34.21 ± 3.29	178.13 ± 24.31	173.41 ± 28.12
Diabetic scrambled siRNA	23.12 ± 2.04	24.36 ± 3.23**	335.16 ± 63.37	388.54 ± 51.46**
GLUT1 siRNA treatment	23.62 ± 2.12	25.23 ± 2.78**	338.42 ± 61.28	380.17 ± 65.81**

**P < 0.01, vs Scrambled siRNA.

### Determination of retinal glucose concentrations

**Figure 1:**
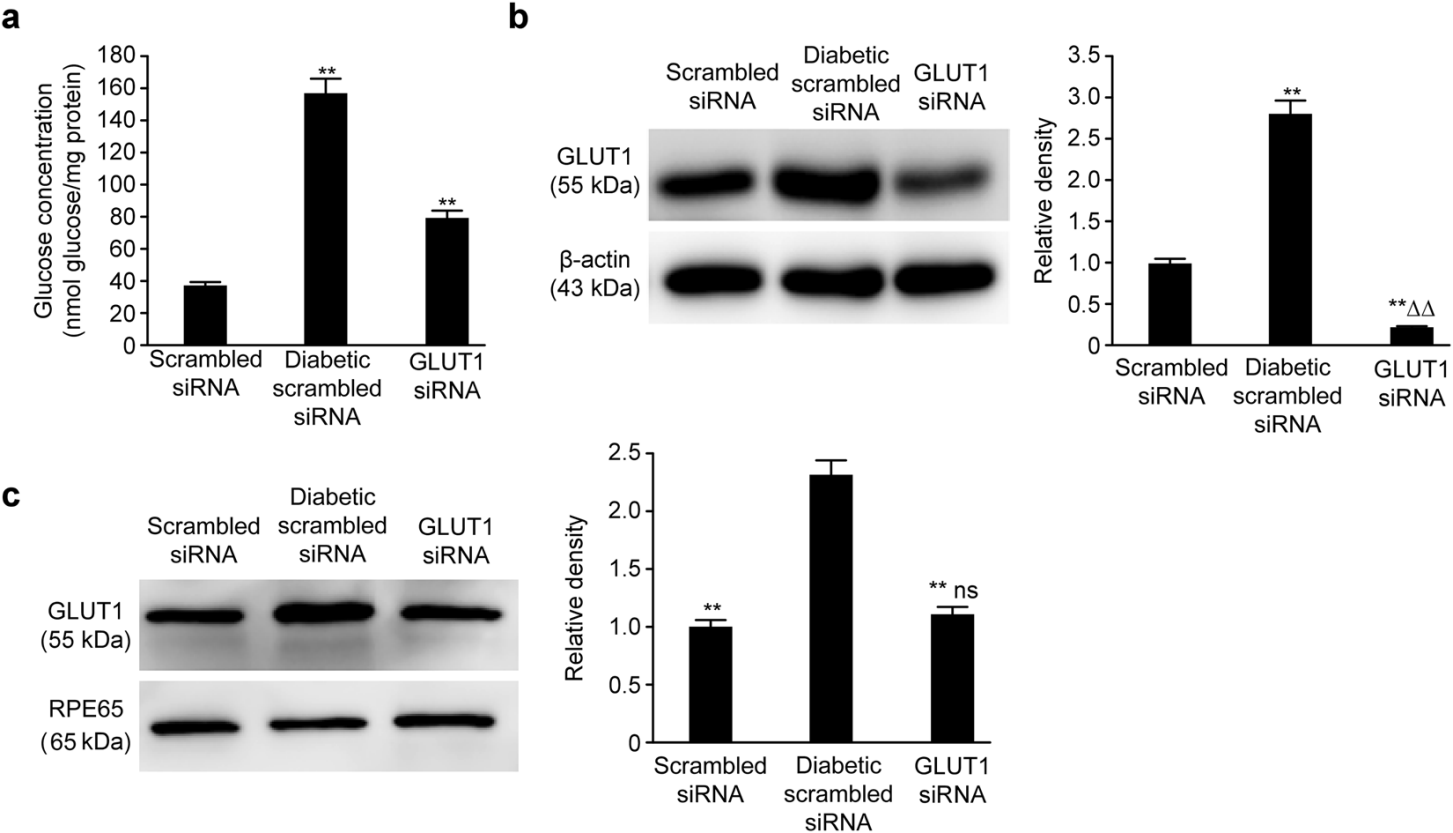
(**a**) Determination of glucose concentration in retinal tissues of the three groups, **P < 0.01 vs. scrambled siRNA group, *n* = 6, $\bar{x}$ ± S. (**b**) GLUT1 expression in the neural retinal layers of the three groups, **P < 0.01 vs. scrambled siRNA group, ^ΔΔ^P < 0.01 vs. diabetic scrambled siRNA group, *n* = 6, $\bar{x}$ ± S. (**c**) GLUT1 expression in the retinal pigment epithelia of the three groups, **P < 0.01 vs. diabetic scrambled siRNA group, ns: P > 0.05 vs. scrambled siRNA group, *n* = 6, $\bar{x}$ ± S. Full-length blots are presented in Supplementary Figure 1

The glucose concentration in the retinal tissue of the scrambled siRNA group was approximately 36.36 ± 2.98 nmol glucose/mg protein, whereas the glucose concentration in the retinal tissue of the diabetic scrambled siRNA group increased to 156.73 ± 8.01 nmol glucose/mg protein at 20 weeks after the diabetic model was established. The glucose concentration in the GLUT1 siRNA group was 78.44 ± 4.96 nmol glucose/mg protein. The glucose concentrations in the retinal tissues of the diabetic model mice of the two groups were significantly higher than those in the mice of the scrambled siRNA group (P < 0.01). However, the glucose concentration in the retinal tissue of the GLUT1 siRNA group was significantly lower than that in the diabetic scrambled siRNA group by 50.05% (P < 0.01) (Fig. 1a).

### Retinal GLUT1 expression in the three groups

Immunoblotting revealed that the expression of GLUT1 in the neural retinal layer was upregulated under diabetic conditions, but the expression of retinal GLUT1 in the GLUT1 siRNA group was lower than that in the scrambled siRNA group by approximately 77.00%; however, GLUT1 expression in the GLUT1 siRNA group was only lower than that in the diabetic scrambled siRNA group by 8.07%. Both of these differences were statistically significant (P < 0.01) (Fig. 1b). Simultaneously, GLUT1 expression in the retinal pigment epithelium was also detected, and the results were different from those obtained in the neural retinal layer. Although GLUT1 expression in the GLUT1 siRNA group was only 50.22% of that in the diabetic scrambled siRNA group, which represented a significant difference (P < 0.01), there was no significant difference compared with that in the scrambled siRNA group (P > 0.05) (Fig. 1c).

### Pathological changes in cone photoreceptors

**Figure 2:**
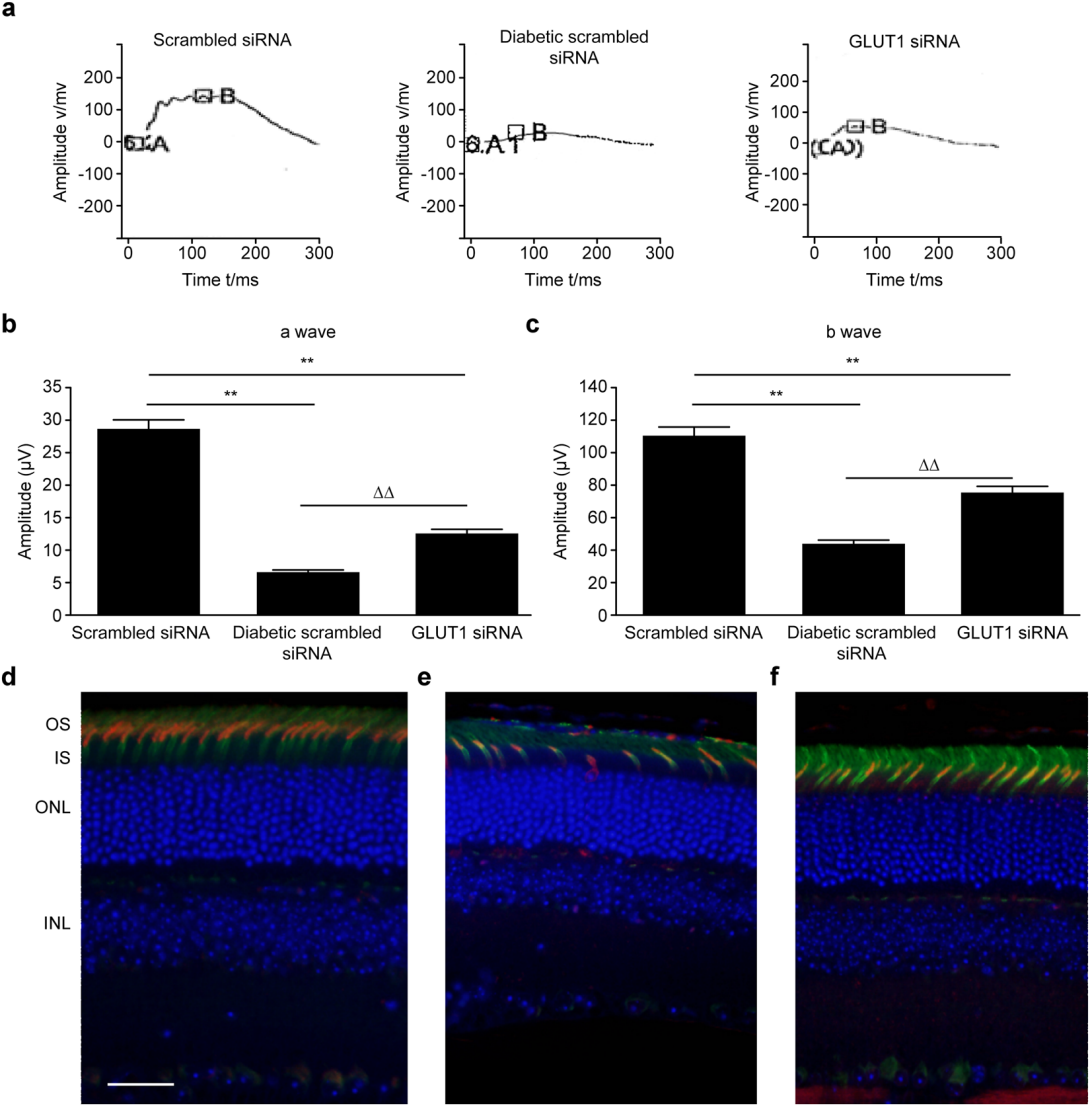
(**a**) Representations of classic photopic ERG waveforms. Figure 2b and c: Results of the statistical analysis of photopic ERG *a* wave (**b**) and *b* wave (**c**) amplitudes (*n* = 9) **P < 0.01, compared with scrambled siRNA group, ^ΔΔ^P < 0.01, compared with diabetic scrambled siRNA group. Figure 2d–f: Changes in cone cells of the three groups were detected using an immunofluorescence colocalization method (fluorescence microscope ×400) (**d**) scrambled siRNA group, scale bar represents 50 µm; (**e**) diabetic scrambled siRNA group; (**f**) GLUT1 siRNA group; green: PNA, red: opsin, blue: DAPI; OS: outer segment of cone cell; IS: inner segment of cone cell.

Photopic electroretinogram amplitudes reflect the function of cone photoreceptors^9^. The photopic ERG *a* and *b* wave amplitudes of both the diabetic scrambled siRNA and GLUT1 siRNA groups were significantly lower than those of the scrambled siRNA group (P < 0.01). However, the photopic ERG *a* and *b* wave amplitudes of the GLUT1 siRNA group were significantly higher than those of the diabetic scrambled siRNA group (Fig. 2a–c). Cone photoreceptors were detected using an immunofluorescence colocalization method. Compared with the scrambled siRNA group, both the diabetic scrambled siRNA and GLUT1 siRNA groups exhibited decreased cone cell density and loosely arranged cones. The changes were more significant in the diabetic scrambled siRNA group, and the cone outer segments in the diabetic scrambled siRNA group appeared shorter than those in the GLUT1 siRNA treatment group (Fig. 2d–f).

### Pathological changes in rod cells

**Figure 3:**
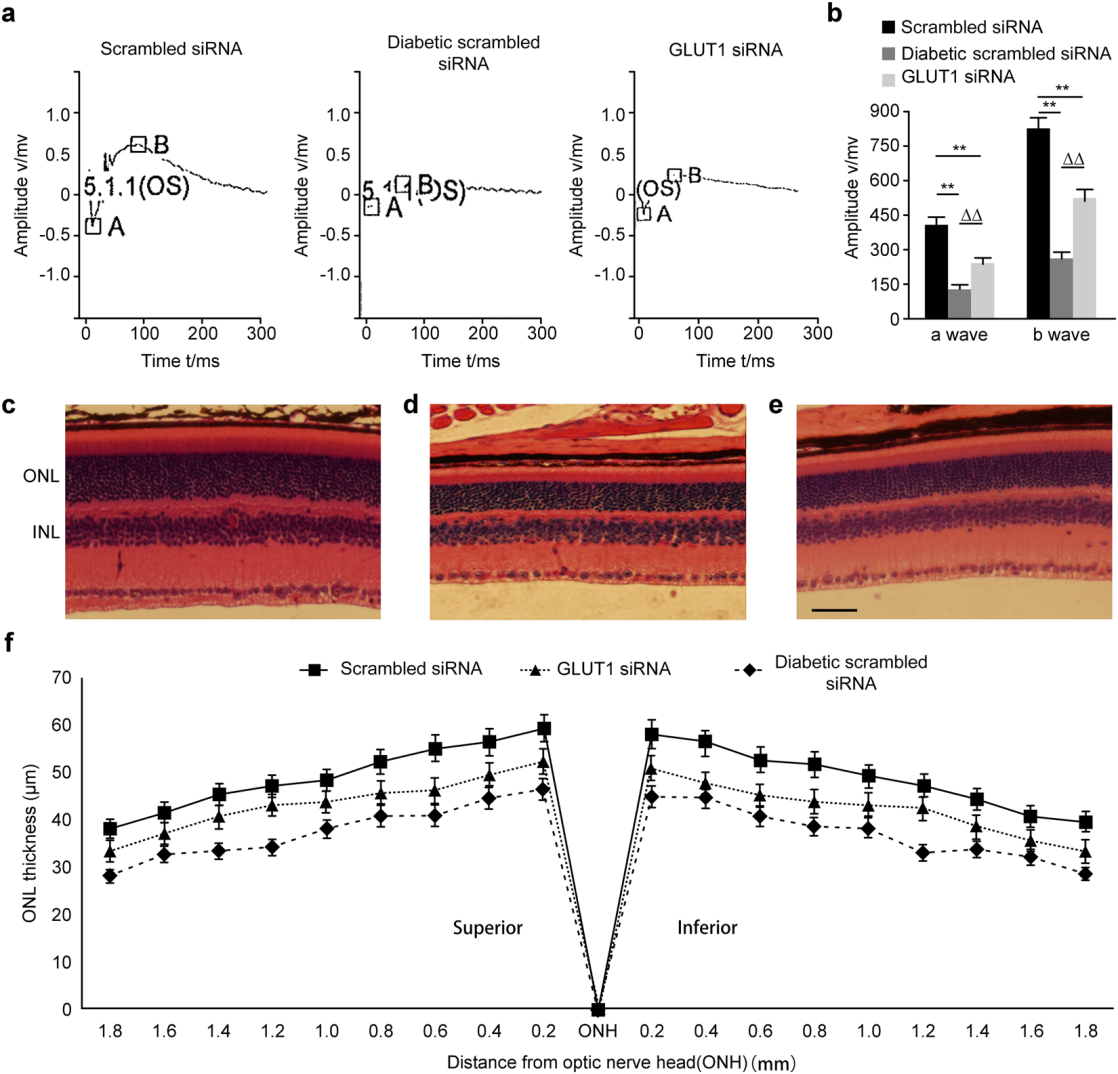
(**a**) Representations of classic scotopic ERG waveforms. Figure 3b: Results of the statistical analysis of scotopic ERG *a* wave and *b* wave amplitudes (*n* = 9) **P < 0.01, compared with scrambled siRNA group, ^ΔΔ^P < 0.01, compared with diabetic scrambled siRNA group. Figure 3c–f: ONL thicknesses of the three groups (inverted microscope ×400) (**d**) scrambled siRNA group; (**e**) diabetic scrambled siRNA group, scale bar represents 50 μm; (**f**) GLUT1 siRNA group; (**f**) Statistical analysis of ONL thicknesses.

Scotopic ERG uses a gradient of luminance to stimulate the retina under dark conditions, which reflects rod cell function^9^. The ERGs of the three groups are shown in Fig. 3a,b; the scotopic ERG *a* and *b* wave amplitudes in both the diabetic scrambled siRNA and GLUT1 siRNA groups were significantly lower than those in the scrambled siRNA group (P < 0.01). However, the scotopic ERG *a* and *b* wave amplitudes in the GLUT1 siRNA group were significantly higher than those in the diabetic scrambled siRNA group. The ONL is primarily composed of photoreceptor nuclei, and ONL thickness essentially reflects changes in the number of rod photoreceptors because rods constitute 98% of all photoreceptors. In our study, ONL thicknesses were measured at 0.48, 0.96, 1.44, and 1.92 mm from the optic nerve. At 20 weeks after the model was established, the ONL thicknesses in the GLUT1 siRNA treatment and diabetic scrambled siRNA groups were lower than those in the scrambled siRNA group by approximately 16.05% and 35.38%, respectively. However, the ONL thicknesses in the GLUT1 siRNA treatment group were significantly thicker than those in the diabetic scrambled siRNA group by 29.92% (P < 0.05) (Fig. 3c–f).

### Inflammatory reactions in the retina

**Figure 4:**
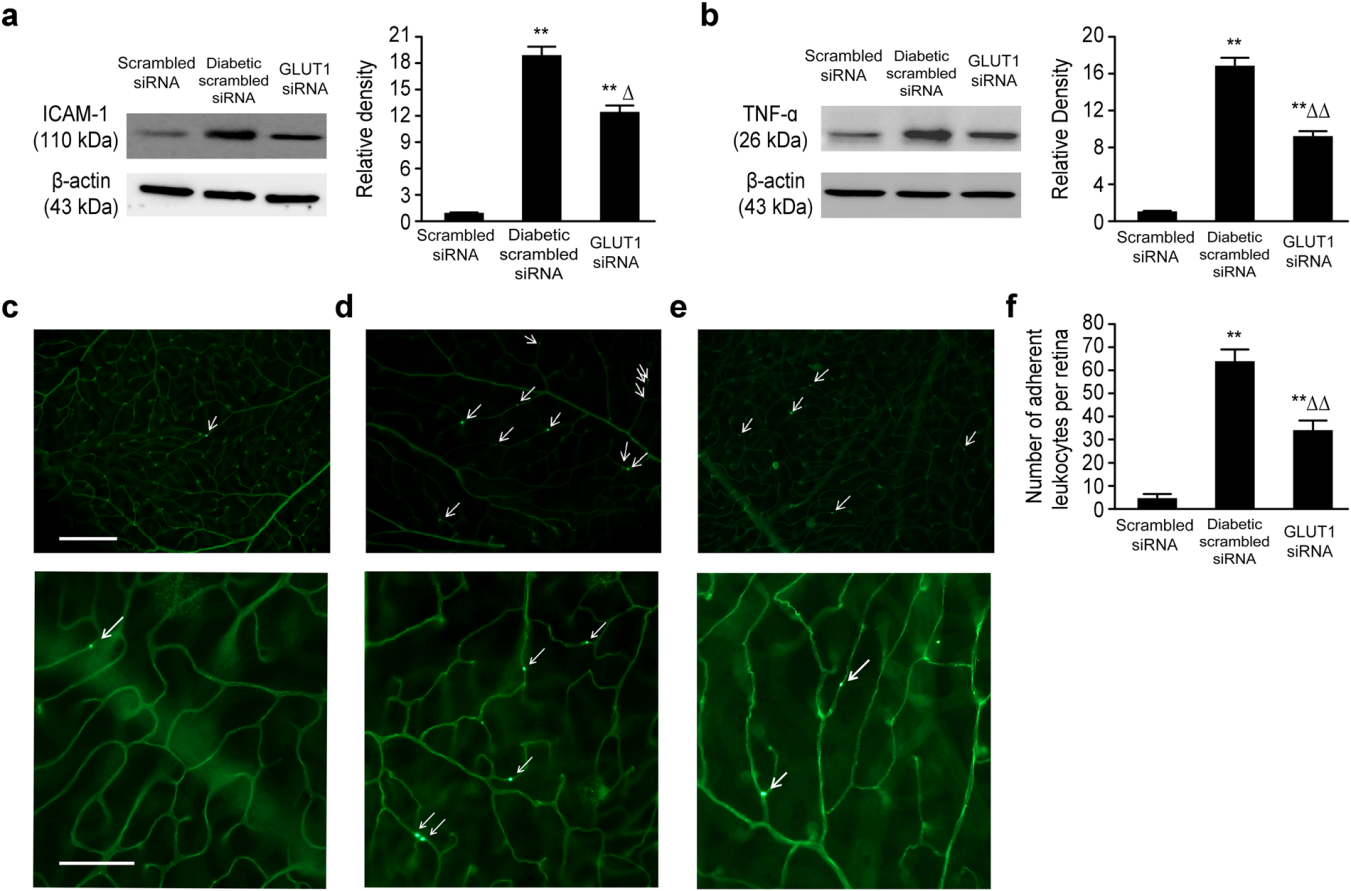
Inflammatory reactions in the retina of mice in the three groups. (**a**) Expression of retinal inflammation marker ICAM-1 in mice of the three groups **P < 0.01 vs. scrambled siRNA group, ^Δ^P < 0.05 vs. diabetic scrambled siRNA group, *n* = 6, $\overline{x}$ ± S. (**b**) Expression of retinal inflammation marker TNF-α in mice of the three groups, **P < 0.01 vs. scrambled siRNA group, ^ΔΔ^P < 0.01 vs. diabetic scrambled siRNA group, *n* = 6, $\overline{x}$ ± S, (**c–f**) Leukocytes adhesion to retinal vessel (**c**): scrambled siRNA group, scale bar represents 100 µm (upper row images)/scale bar represents 50 µm (lower row images); (**d**) diabetic scrambled siRNA group; (**e**) GLUT1 siRNA group; white arrows indicates adherent leukocytes; (**f**) statistical analysis **P < 0.01 vs. scrambled siRNA group, ^ΔΔ^P < 0.01 vs. diabetic scrambled siRNA group, *n* = 6, $\overline{x}$ ± S (fluorescence microscope ×400). Full-length blots are presented in Supplementary Figure 2.

Previous research has demonstrated that DR is an inflammatory disease^10^, and ICAM-1 and TNF-α are two important inflammation markers. Immunoblotting revealed that the expression levels of ICAM-1 in the diabetic scrambled siRNA and GLUT1 siRNA groups were significantly upregulated compared with those in the scrambled siRNA group (P < 0.01). However, the expression of retinal ICAM-1 in the GLUT1 siRNA group was approximately 66.14% of that in the diabetic scrambled siRNA group (P < 0.05) (Fig. 4a). Similar results were obtained for the expression levels of TNF-α, which were also significantly upregulated in both the diabetic scrambled siRNA and GLUT1 siRNA groups compared with those in the scrambled siRNA group (P < 0.01). However, the expression of retinal TNF-α in the GLUT1 siRNA group was approximately 54.76% of that in the diabetic scrambled siRNA group (P < 0.05) (Fig. 4b).

Leukostasis is also an important indicator of retinal inflammatory reactions^9^, as well as early pathological changes in DR. No adherent leukocytes were found in the scrambled siRNA group, whereas adherent leukocytes were detected in the diabetic scrambled siRNA and GLUT1 siRNA groups. However, the number of adherent leukocytes in the GLUT1 siRNA group was approximately 52.76% of that in the diabetic scrambled siRNA group (P < 0.01) (Fig. 4a–d).

### Blood–retinal barrier leakage

**Figure 5:**
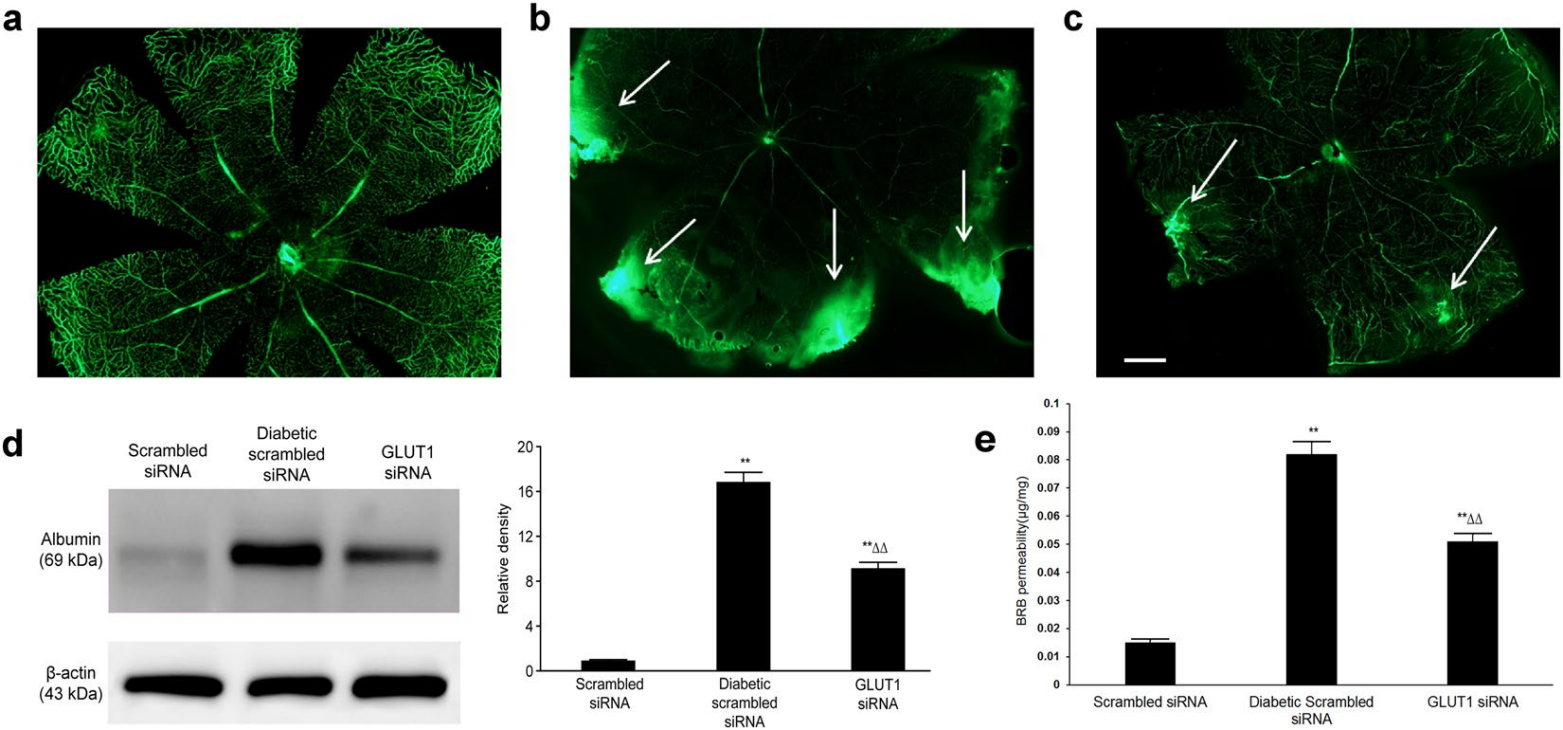
Comparison of leakage of the inner blood–retinal barrier among the three groups (**a**) scrambled siRNA group; (**b**) diabetic scrambled siRNA group; (**c**) GLUT1 siRNA group, scale bar represents 200 µm, fluorescence microscope ×400; white arrows indicate fluorescence leakage regions; (**d**) Expression of retinal albumin in mice of the three groups, (**e**) BRB permeability assay using Evans blue dye in mice of the three groups, **P < 0.01 vs. scrambled siRNA group, ^ΔΔ^P < 0.01 vs. diabetic scrambled siRNA group, *n* = 6, $\overline{x}$ ± S. Full-length blot is presented in Supplementary Figure 3.

We used fluorescence microscopy to observe and compare fluorescein isothiocyanate-labeled bovine serum albumin as measurement of inner blood–retinal barrier leakage. The results showed that the inner blood–retinal barrier in the scrambled siRNA group was intact, and no fluorescence leakage was observed, whereas fluorescence leakage regions were detected in the diabetic scrambled siRNA and GLUT1 siRNA groups. However, fewer fluorescence leakage regions and smaller leakage areas were found in the GLUT1 siRNA group than in the diabetic scrambled siRNA group (Fig. 5a–c). We also used immunoblotting to measure the content of retinal albumin, and the albumin expression levels were also significantly increased in both the diabetic scrambled siRNA and GLUT1 siRNA groups compared with those in the scrambled siRNA group (P < 0.01). However, the expression of retinal albumin in the GLUT1 siRNA group was approximately 56.18% of that in the diabetic scrambled siRNA group (P < 0.01) (Fig. 5d). As shown in Fig. 5e, BRB permeability was also measured *in vivo* using the Evans blue dye. The concentration of Evans blue in formamide extract of diabetic retina was significantly higher than scrambled siRNA group (P < 0.01). GLUT1 siRNA treatment significantly reduced Evans blue extravasation compared to diabetic scrambled siRNA group (P < 0.01).

## Discussion

DR is the one of the most common and serious ocular complications, and its pathogenesis remains unclear. The key effects of high blood glucose levels in DR and other diabetes-related complications have been demonstrated in the clinical trials DCCT^2^ and UKPDS^3^. The effects of high blood glucose on retinal cells may include changes in the expression levels of specific genes, buildup of advanced glycation end products, and increased oxidative stress reactions^11^. Given that the high-glucose microenvironment in DR damages retinal tissues, controlling the glucose content of local retinal tissues and reversing the high-glucose microenvironment may address the problem. However, glucose in the retina is transferred from blood circulation and cannot pass through the phospholipid bilayer of mammalian cell membranes due to its water solubility; thus, GLUT, a family of transport proteins, is used to transport glucose^12^, which is required for retinal tissues to take up glucose: GLUT1 is the only carrier for the transport of glucose across the blood–retinal barrier^7^.

Researches concerning GLUT1 expression under high glucose condition are contrary at present. Kumagai *et al*. examined GLUT1 expression in the eyeballs (without or with mild retinopathy) of patients with diabetes using immunocytochemistry and found that the activity of retinal GLUT1 in more than half of the eyeballs was 18 times higher than that in the eyeballs of the normal control group^13^. Fernandes *et al*. found that there was no compensatory downregulation of GLUT1 on the inner BRB in diabetic rats by means of immunogold staining^14^. However, Fernandes *et al*. also reported that GLUT1 expression was decreased in alloxan-induced diabetic rabbits^15^. Similarly, Badr *et al*. suggested that diabetic condition downregulated GLUT1 expression in the retina and its microvessels^16^. These controversial results may attribute to different animal species, diabetic course and methodology. In our study, GLUT1 level in diabetic scrambled siRNA group was 2.67 times that of scrambled siRNA group.

siRNA is a type of RNA fragment that ranges in size from 19 bp to 21 bp. siRNA can specifically degrade mRNA of particular genes to inhibit the expression of these genes. In our experiment, we administered effective GLUT1 siRNA sequences, which were identified in previous studies^17^, to decrease the amount of glucose transported into the retina. As mentioned above, no significant difference in the overall blood glucose levels was found between the mice with diabetes of both groups at 20 weeks after the diabetic model was established. GLUT1 siRNA was intravitreally injected into mice of the GLUT1 siRNA group, and the expression of retinal GLUT1 was downregulated accordingly: it was decreased by approximately 91.93% compared with that in the diabetic scrambled siRNA group and by approximately 77% compared with that in the scrambled siRNA group. At the same time, the retinal glucose concentration in the GLUT1 siRNA group was only 50.05% of that in the diabetic scrambled siRNA group. This finding indicates that the amount of glucose transported into the retina was effectively reduced after GLUT1 was inhibited by GLUT1 siRNA. The retinal glucose concentration in the GLUT1 siRNA group remained higher than that in the scrambled siRNA group by approximately 53.64% because intravitreal injections of GLUT1 siRNA significantly inhibited GLUT1 within the inner blood–retinal barrier. However, GLUT1 siRNA had a limited effect on the retinal pigment epithelium, which forms the outer blood–retinal barrier, and the expression of GLUT1 in the retinal pigment epithelium was not downregulated. The biological activities of GLUT1 have also been found to be upregulated under diabetic conditions compared with those under normal conditions^18, 19^. Consequently, the retinal glucose transported from the outer blood–retinal barrier resulted in higher retinal glucose concentrations in the GLUT1 siRNA group than those in the scrambled siRNA group. Therefore, we established the conditions predicted in our hypothesis by restricting GLUT1 in the inner blood–retinal barrier. Next, we determined if the function and morphology of photoreceptors and the level of microangiopathy were affected using various indicators.

Non-invasive recording is performed in ERG via platinum electrodes at the surface of cornea, using flashes of different brightness to stimulate the electrical activity of photoreceptor cells^9^. Scotopic ERG and photopic ERG are often used to measure the function of rod photoreceptors and cone photoreceptors, respectively. Previous studies have shown that abnormal ERGs are detected at the early stage of diabetes in rats^20^. Another study has reported that functional disorder of retinal photoreceptors occurs in DR patients at the non-proliferative phase before the onset of microangiopathic lesions, such as fundus neovascularization, when inspected using flash ERG^21^. In our study, although the scotopic ERG and photopic ERG *a* and *b* wave amplitudes in the GLUT1 siRNA group were lower than those in the scrambled siRNA group, they were higher than those in the diabetic scrambled siRNA group. This finding indicates relatively mild functional impairment in photoreceptor cells of mice with diabetes after glucose transport into the retina was restricted by GLUT1 siRNA.

ONL thickness was primarily used to measure rod photoreceptors, and PNA was used to label cone photoreceptors. Another study has shown that the ONL thickness of diabetic rats was gradually decreased over the course of the illness^20^. Researchers have recognized that the degeneration and death of rod cells are the primary cause of abnormal visual function in patients with diabetes before the presentation of DR and the associated important pathological changes^22^. Moreover, in this study, the ONL thicknesses in diabetic model mice of both groups were lower than those in mice of the scrambled siRNA group at 20 weeks after the diabetic model was established. This finding indicates that the rod cells in the diabetic model mice were constantly dying throughout the experiment. However, the ONL thickness at each time point in the GLUT1 siRNA group was higher than that in the diabetic scrambled siRNA group. In addition to PNA labeling, we also used S-opsin to mark the outer segments of cone cells^23^ and found that the cone cells were more loosely arranged and had shorter outer segments in the diabetic scrambled siRNA group than those in the GLUT1 siRNA group, as previously described. The above results suggest that although photoreceptor cells were constantly dying under diabetic conditions, the numbers of dead rod and cone cells in the GLUT1 siRNA treatment group were relatively low, which also demonstrates the protective effect of a relatively low blood glucose microenvironment on photoreceptor cells.

Inflammatory reactions are an important process in the microangiopathy of DR; numerous studies have indicated that the number of retinal leukocytes with enhanced adhering ability and decreased deformability^24, 25^ is increased in diabetic animal models. Adherent leukocytes increase due to reduced passive deformability when passing through capillary vessels with sizes less than the diameter of the leukocytes in DR patients; adherent leukocytes also significantly increase in number throughout the progression of DR^26^. Therefore, a leukostasis assay can be used for the analysis of inflammatory reaction levels in DR. In our study, although the number of adherent leukocytes in retinal vessels in the GLUT1 siRNA group was higher than that in the scrambled siRNA group, it was only 52.76% of the total number detected in the diabetic scrambled siRNA group. We detected the expression levels of two inflammation markers simultaneously, including chemotactic factor ICAM-1 and cytokine TNF-α. ICAM-1 and its ligand CD18 play an important role in mediating leukocyte adhesion^27^, and inhibition of ICAM-1 results in significant mitigation of leukocyte adhesion and vasopermeability^28^. Expression of TNF-α is also upregulated in the retina under DR conditions^29^. The expression levels of both inflammatory factors in the retina of the GLUT1 siRNA group were only 66.14% and 54.76% of those in the diabetic scrambled siRNA group. This finding indicates a relatively mild inflammatory reaction in mice with diabetes after glucose transport into the retina was restricted by GLUT1 siRNA. Damage to the blood–retinal barrier is an important cause of retinal edema, particularly macular edema, which might be ascribed to the increase in leukostasis and upregulation of inflammation marker expression^9^. As described above, the numbers of adherent leukocytes and levels of inflammation factors in the GLUT1 siRNA group were significantly lower than those in the diabetic scrambled siRNA group. When we examined the leakage of the inner blood–retinal barrier, we identified fewer leakage regions and smaller leakage areas in the GLUT1 siRNA group compared with those in the diabetic scrambled siRNA group. Similar result was obtained by Evans blue permeation assay. These findings indicate that the relatively low blood glucose microenvironment of the retina exerted a protective effect on the inner blood–retinal barrier.

In summary, after an intravitreal injection of GLUT1 siRNA was administered to inhibit GLUT1 in the retina, the retinal glucose concentration in mice with diabetes was decreased. Therefore, a retinal microenvironment with relatively low glucose levels was formed. Under this environment, pathological changes in the function and morphology of retinal photoreceptors and the pathological changes associated with microangiopathy were relieved to some extent compared with those in mice with diabetes, which suggests that restricting local retinal glucose content by inhibiting GLUT1 might be a new direction for the prevention and treatment of DR in the future.

## Materials and Methods

### Synthesis of GLUT1 siRNA

An effective siRNA sequence was designed according to reference^17^, and Shanghai GenePharma Company synthesized the GLUT1-targeted siRNA (positive-sense strand 5′-GGAATTCAATGCTGATGATGA-3′ and antisense strand 5′-TCATCATCAGCATTGAATTCC-3′) and the non-targeted siRNA as a negative control (positive-sense strand 5′-TTCTCCGAACGTGTCACGT-3′ and antisense strand 5′-ACGTGACACGTTCGGAGAA-3′). Normal saline treated with diethy pyrocarbonate (Sigma-Aldrich Corp. St. Louis, MO, USA.) was used to dissolve siRNA to reach a 20 μmol/L concentration.

### Experimental animals and grouping

This study was carried out in strict accordance with the recommendations in the Guide for the Care and Use of Laboratory Animals of the National Institutes of Health. The protocol was approved by the Committee on the Ethics of Animal Experiments of Nanchang University. All surgeries were performed under ketamine & xylazine anesthesia, and all efforts were made to minimize suffering. A total of 48 male inbred line C57BL/6 mice at eight weeks of age without eye diseases and weighing 20 g to 30 g were purchased from the Animal Science Department, Nanchang University. After we marked ear nails and serial numbers for the mice, the animals were randomly divided into scrambled siRNA, diabetic scrambled siRNA, and GLUT1 siRNA treatment groups, with 16 mice in each group. Establishment of DM model: Streptozotocin (Sigma-Aldrich Corp. St. Louis, MO, USA.) was intraperitoneally injected into mice for 5 consecutive days after the mice fasted for 8 h. Streptozotocin (50 mg/kg body weight in 0.01 mol/L citrate buffer solution [pH 4.5]) was intraperitoneally injected into the diabetic scrambled siRNA and GLUT1 siRNA groups, whereas an equal amount of citrate buffer solution was injected into the scrambled siRNA group. A blood sample was collected from the caudal vein to measure blood glucose levels at 7 d. The standard for successful establishment of the DM model was a blood glucose level > 300 mg/dL.

### Intravitreal injection with siRNA

We performed intravitreal injections in the first week after diabetes induction. Intraperitoneal anesthesia with mixture of ketamine and xylazine (Sigma-Aldrich Corp. St. Louis, MO, USA.) was administered to the three groups and iodophor disinfection was conducted around the eyes subsequently. A thirty-Gauge needle (Becton, Dickinson and Company. Franklin Lakes, NJ, USA.) was inserted using a Hamilton microinjector (Hamilton Company, Reno, NV, U.S.A) toward the optic nerve at 1 mm outside of the limbus under a microscope. The medicine was slowly injected after the needle tip was detected in the pupil area. A volume containing 1 μL of 20 μmol/L GLUT1 siRNA and 1 μL of transfection reagent was intravitreally injected into the GLUT1 siRNA treatment group, whereas a volume containing 1 μL of 20 μmol/L non-targeted siRNA and 1 μL of transfection reagent (Invitrogen, Waltham, MA, USA) was intravitreally injected into the scrambled siRNA and diabetic scrambled siRNA groups. The injection was conducted in both eyes and repeated twice a week until nine injections were completed.

### Electroretinography

Electroretinography was inspected at 20 weeks after the DM model was established. All mice were dark-adapted overnight in a dark chamber after pupil dilation was induced by tropicamide eye drops (Santen Pharmaceutical Co., Ltd. Kita-ku, Osaka, Japan). Anesthesia, consisting of ketamine and xylazine, was administered the next day. The mice were then placed on a heating board. The reference and ground electrodes were inserted into the palate and tail, respectively. Platinum corneal electrodes were placed on cornea of both eyes, and recombinant bovine fibroblast growth factor eye gel was applied for lubrication. Mouse ERG preparation was completed under dim red lighting in the dark chamber. Illumination intensities of 0.0004, 0.04, 4, 400, and 2000 cd•s/m^2^ were used to record scotopic ERG by Espion electroretinogram E2 system (Diagnosys, Lowell, MA, USA) Then, the mice were light adapted for 10 min, and photopic ERGs were recorded under an illumination intensity of 2000 cd•s/m^2^.

### Determination of retinal glucose concentrations

Six eyeballs were enucleated for measurement of retinal glucose concentrations. Retinal tissues were collected, and 50 μL of deionized water was added to the tissues. Samples were heated at 70 °C for 15 min, followed by ultrasonication for 30 s, and centrifugation for 20 min. Up to 35 μL of supernatant was transferred into 165 μL of reagent of a glucose concentration assay kit (Sigma-Aldrich Corp. St. Louis, MO, USA.), followed by the establishment of a standard curve and blank control. A spectrum analyzer (SPECTRO Analytical Instruments GmbH, Boschstr, Kleve, Germany) was used to measure the optical density of the samples, and SPECTROstar Nano MARS software (SPECTRO Analytical Instruments GmbH, Boschstr, Kleve, Germany) was used to calculate glucose concentrations. Subsequently, 10 μL of supernatant was added to 190 μL of reagent of a protein concentration assay (BIO-RAD Laboratories, Inc., Hercules, CA, USA). A standard curve and blank control were also established. The spectrum analyzer was used to measure the optical density of the samples, and SPECTROstar Nano MARS software was used to calculate protein concentrations. Retinal glucose concentration is presented as nmol/mg, and the calculation formula was G × GV/GMW × (P × PV), where G = glucose concentration (ng/mL), GV = volume of liquid used to determine glucose content (mL), P = protein concentration (mg/mL), PV = volume of liquid used to determine protein content (mL), and GMW = glucose molecular weight (180.2).

### ONL thickness measurement

Eyeballs were enucleated and directly fixed in 4% paraformaldehyde for 1 h. The cornea and lens were then removed, and the eyes were fixed again in 4% paraformaldehyde for 15 min. Subsequent steps were performed in accordance with a conventional hematoxylin-eosin staining protocol. Slices were sealed and observed under a microscope. ImagePro software (Olympus Corporation, Tokyo, Japan) was used to measure ONL thickness at 0.2, 0.4, 0.6, 0.8, 1.0, 1.2, 1.4, 1.6 and 1.8 mm from the optic nerve.

### Immunofluorescence colocalization method

Eyeballs were enucleated and directly fixed in 4% paraformaldehyde for 1 h. The cornea and lens were then removed, and the eyes were fixed again in 4% paraformaldehyde for 15 min. Subsequent steps were performed in accordance with a conventional protocol. After paraffin sections were prepared, dewaxing and heat-induced antigen retrieval were performed in accordance with a conventional protocol. The sections were then incubated with S-opsin primary antibodies (Millipore Corporation. St. Charles, MI, USA), followed by incubation with Peanut agglutinin (PNA) (Vector Laboratories., Burlingame, CA, USA) secondary antibodies the next day. After DAPI (Vector Laboratories., Burlingame, CA, USA) was added, the slices were observed under a fluorescence microscope.

### Immunoblotting

Six eyeballs were enucleated, and retinal tissues were collected and placed into Eppendorf tubes with 200 μL of lysate. The remaining tissues- “eyecups” were also mounted in tissue culture plate (Corning Incorporated, Corning, NY, USA) and up to 5 μL of lysate was added into the eyecups to extract retinal pigment epithelial proteins. After 5 min, the lysates were collected. Subsequent steps were performed in accordance with a conventional protocol. Equal amounts of protein samples were used for SDS-PAGE electrophoresis and transmembrane incubation with GLUT-1 (Millipore Corporation. St. Charles, MI, USA), ICAM-1, TNF-α (Santa Cruz Biotechnology, Inc., Dallas, TX, USA) and albumin (Abcam plc, Cambridge, UK) primary antibodies. The following day, secondary antibody incubation was conducted at room temperature for 1 h after the membranes were washed three times. Finally, the relative densities of the blots were measured by UVP GelDoc-It Imager (UVP LLC, Upland, CA, USA).

### Leukostasis assay

Anesthesia, consisting of ketamine and xylazine, was administered to three mice from each of the three groups. The chest skin and ribs were cut open to expose the thoracic cavity. The descending aorta was closed by clamping, and the right auricle was cut open. A 27 G needle was inserted into left ventricle. Initially, 10 mL of PBS with heparin (0.1 mg/mL) was used to perfuse the tissue and remove leukocytes that did not adhere to retinal vessels. An additional volume of 20 μg/mL of PBS and FITC- Concanavalin A (5 mg/kg) (Sigma-Aldrich Corp. St. Louis, MO, USA.) was used to label adherent leukocytes in retinal vessels. Up to 10 mL of PBS was reused to remove excess FITC- Concanavalin A. The flow rate of perfusion is 3–4 ml/min. Six eyeballs were enucleated and directly fixed in 4% paraformaldehyde for 1 h. Retinal flat mounts were prepared, and a fluorescence microscope was used to observe and quantify the total number of adherent leukocytes in the whole retina.

### Blood–retinal barrier leakage

Ketamine and xylazine were used to anesthetize three mice from each of the three groups. FITC-BSA (66 kDa, 100 mg/kg) (Sigma-Aldrich Corp. St. Louis, MO, USA.) was injected into the femoral vein. The mice were killed after 20 min, and six eyeballs from each group were enucleated and fixed in 4% paraformaldehyde for 30 min. Retinal whole-mounts were prepared, and blood–retinal barrier leakage was observed under a fluorescence microscope.

### Evans blue dye assay

Mouse was injected with received Evans blue dye (45 mg/kg) (Sigma Aldrich, St. Louis, MO, USA) via the tail vein. After 2 hours, 0.2 mL of blood sample was drawn from re-anesthetized mice, and mouse were perfused via the left ventricle with 200 mL PBS to wash out dye. Retina was dissected out and treated with dimethylformamide (Sigma Aldrich, St. Louis, MO, USA) overnight at 70 °C for 18 hours. The extract was centrifuged for 45 min. A spectrum analyzer (SPECTRO Analytical Instruments GmbH, Boschstr, Kleve, Germany) was used to test supernatant at 620 nm and 740 nm. Blood samples were centrifuged for 15 min and the supernatant was diluted 1:1000. The concentration of Evans blue in the blood and retina was used to assess BRB breakdown.

### Statistical analysis

Statistical software SPSS17.0 was used to perform analyses. The results are presented as $\bar{x}$ ± S or $\bar{x}$ ± SEM, and chi-square test was used to compare groups. P < 0.05 was considered significant.

## Electronic supplementary material

Supplementary Information
