# Supplementary material for: Suppression of diabetic retinopathy with GLUT1 siRNA
Source: Sci Rep. 2017 Aug 7;7:7437. doi: 10.1038/s41598-017-07942-x (PMC5547104; doi:10.1038/s41598-017-07942-x)
Supplement: Supplementary file 1 — Supplementary Information [file 41598_2017_7942_MOESM1_ESM.pdf]

Supplementary Information

Suppression of diabetic retinopathy with GLUT1 siRNA

Zhi-Peng You[*], Yu-Lan Zhang[*], Ke Shi[#], Lu Shi, Yue-Zhi Zhang, Yue Zhou, Chang-yun Wang

*Zhi-Peng You and Yu-Lan Zhang equally contributed to the work.

[#]**Correspondence**: Ke Shi

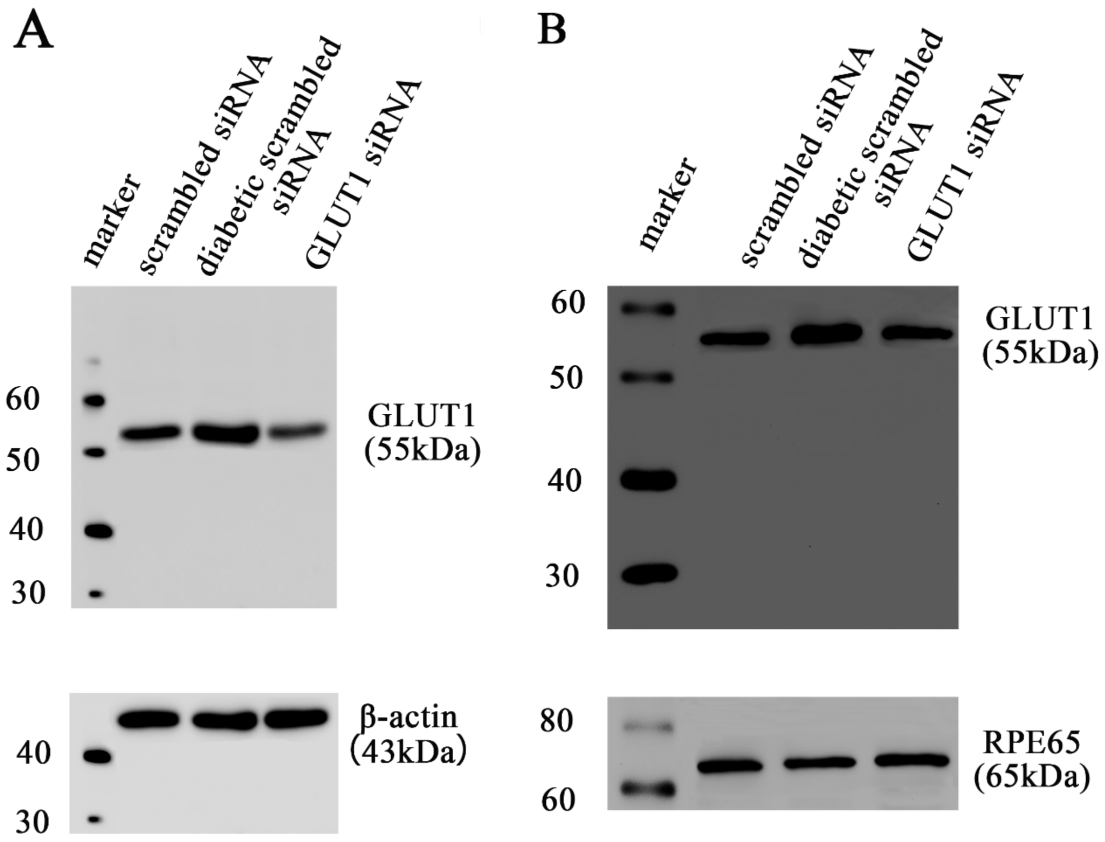

Supplementary Fig. S1 Full Western blots of Fig. 1.

Fig. S1A: full Western blot of Fig. 1 b .

Fig. S1B: full Western blot of Fig. 1 c .

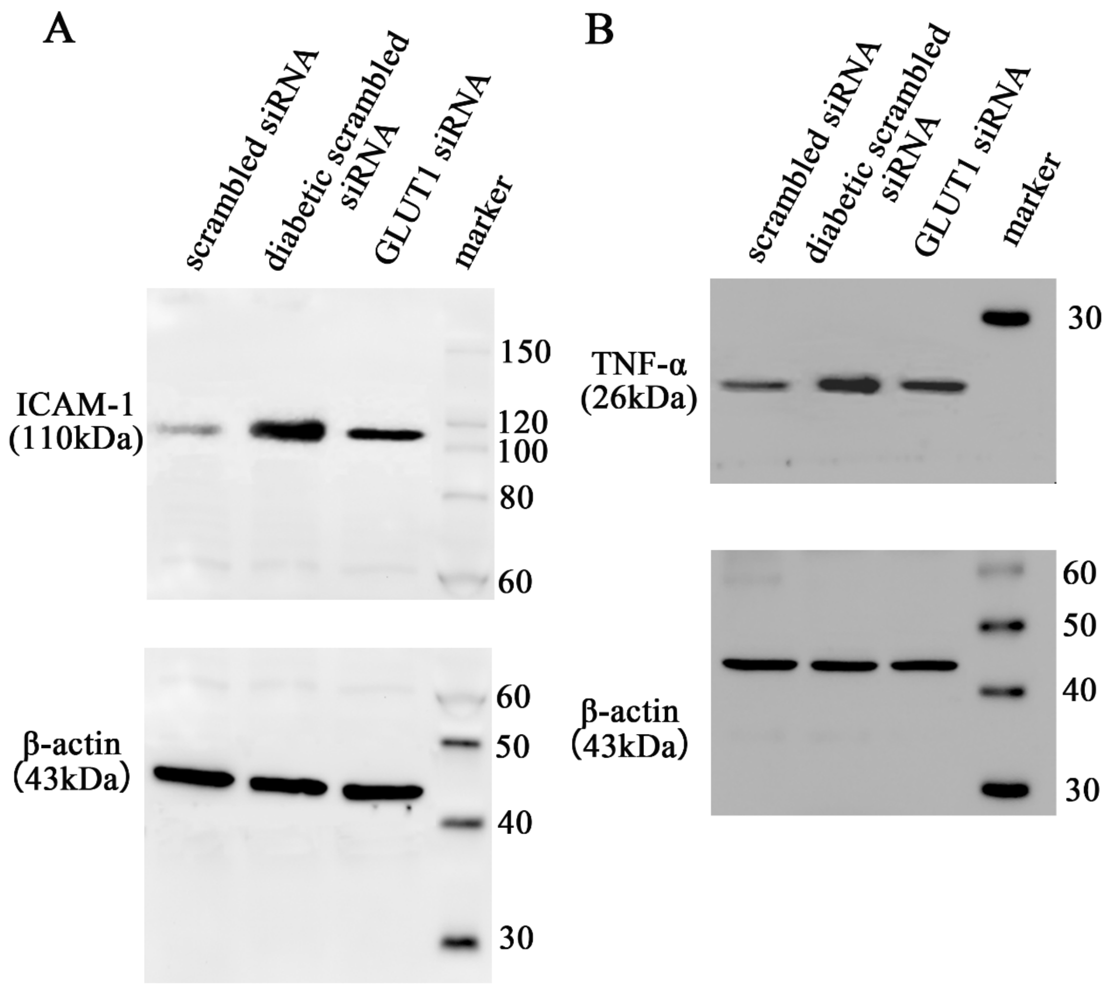

Supplementary Fig. S2 Full Western blots of Fig. 4.

Fig. S2A: full Western blot of Fig. 4 a .

Fig. S2B: full Western blot of Fig. 4 b .

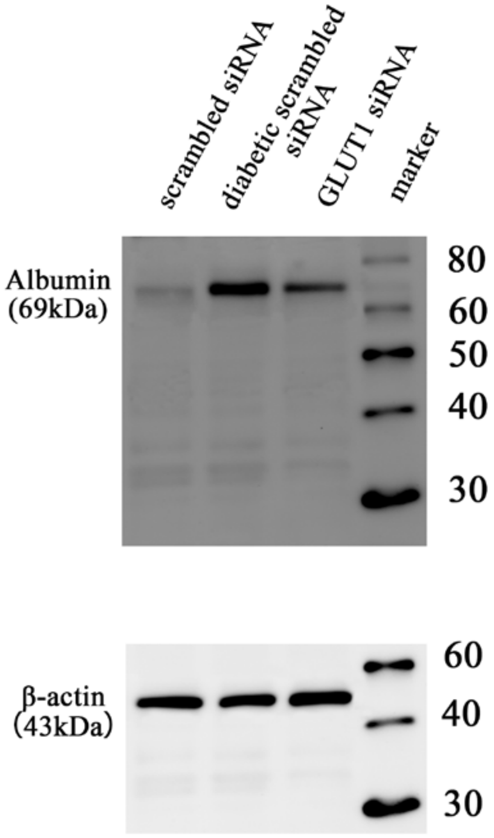

Supplementary Fig. S3 Full Western blots of Fig. 5.
Fig. S3: full Western blot of Fig. 5 d.
